# AmpuBase: a transcriptome database for eight species of apple snails (Gastropoda: Ampullariidae)

**DOI:** 10.1186/s12864-018-4553-9

**Published:** 2018-03-05

**Authors:** Jack C. H. Ip, Huawei Mu, Qian Chen, Jin Sun, Santiago Ituarte, Horacio Heras, Bert Van Bocxlaer, Monthon Ganmanee, Xin Huang, Jian-Wen Qiu

**Affiliations:** 1 HKBU Institute of Research and Continuing Education, Shenzhen, China; 2 Department of Biology, Hong Kong Baptist University, Hong Kong, China; 3 Department of Computer Science, Hong Kong Baptist University, Hong Kong, China; 4 Division of Life Science, The Hong Kong University of Science and Technology, Hong Kong, China; 5 Instituto de Investigaciones Bioquímicas de La Plata (INIBIOLP), Universidad Nacional de La Plata (UNLP)-CONICET CCT-La Plata, La Plata, Argentina; 6 Cátedra de Química Biológica, Facultad de Ciencias Naturales y Museo, UNLP, La Plata, Argentina; 7 Centre national de la recherche scientifique (CNRS), Université de Lille, UMR 8198 – Evo-Eco-Paléo, 59000 Lille, France; 8 Limnology Unit, Department of Biology, Ghent University, 9000 Ghent, Belgium; 9 Department of Animal Production Technology and Fisheries, Faculty of Agricultural Technology, King Mongkut’s Institute of Technology Ladkrabang, Bangkok, 10520 Thailand

**Keywords:** (3 to 10) biological invasion, Caenogastropoda, Genomic database, RNA-Seq, *Lanistes*, *Pila*, *Asolene*, *Marisa*, *Pomacea*

## Abstract

**Background:**

Gastropoda, with approximately 80,000 living species, is the largest class of Mollusca. Among gastropods, apple snails (family Ampullariidae) are globally distributed in tropical and subtropical freshwater ecosystems and many species are ecologically and economically important. Ampullariids exhibit various morphological and physiological adaptations to their respective habitats, which make them ideal candidates for studying adaptation, population divergence, speciation, and larger-scale patterns of diversity, including the biogeography of native and invasive populations. The limited availability of genomic data, however, hinders in-depth ecological and evolutionary studies of these non-model organisms.

**Results:**

Using Illumina Hiseq platforms, we sequenced 1220 million reads for seven species of apple snails. Together with the previously published RNA-Seq data of two apple snails, we conducted de novo transcriptome assembly of eight species that belong to five genera of Ampullariidae, two of which represent Old World lineages and the other three New World lineages. There were 20,730 to 35,828 unigenes with predicted open reading frames for the eight species, with N50 (shortest sequence length at 50% of the unigenes) ranging from 1320 to 1803 bp. 69.7% to 80.2% of these unigenes were functionally annotated by searching against NCBI’s non-redundant, Gene Ontology database and the Kyoto Encyclopaedia of Genes and Genomes. With these data we developed AmpuBase, a relational database that features online BLAST functionality for DNA/protein sequences, keyword searching for unigenes/functional terms, and download functions for sequences and whole transcriptomes.

**Conclusions:**

In summary, we have generated comprehensive transcriptome data for multiple ampullariid genera and species, and created a publicly accessible database with a user-friendly interface to facilitate future basic and applied studies on ampullariids, and comparative molecular studies with other invertebrates.

**Electronic supplementary material:**

The online version of this article (10.1186/s12864-018-4553-9) contains supplementary material, which is available to authorized users.

## Background

Apple snails are a family (Ampullariidae) of snails belonging to Caenogastropoda, the largest and most diverse clade within the class Gastropoda [1–3]. Apple snails seem to have originated on Gondwana [4], with the oldest fossils coming from Early Cretaceous deposits in Africa [5]. After the breakup of Gondwana roughly 100 million years ago, apple snails have undergone diversification in the New World and Old World respectively [4, 6]. Currently, around 120 species of apple snails are recognised in nine genera, including the Old World genera *Afropomus*, *Forbesopomus*, *Lanistes*, *Pila* and *Saulea*, and the New World genera *Asolene*, *Felipponea*, *Marisa* and *Pomacea* [7]. In what follows we abbreviate *Pomacea*, but not *Pila* to avoid confusion of these two genera. Ampullariids are distributed in a wide variety of freshwater habitats, including swamps, wetlands, lakes and rivers [7–9]. Members of the family exhibit a wide range of morphological, behavioural and physiological adaptations to their inhabited environments [10, 11]. For example, the evolutionary radiation of *Lanistes* in Lake Malawi contains species with contrasting morphological and behavioural features that have been interpreted as differential adaption to habitats which differ in wave action, food resources, and predators [9, 12]. Due to their long evolutionary history, wide geographic distribution and high diversity, Hayes et al. [4] suggested that ampullariids altogether provide an interesting system to study speciation and phylogeography in freshwater gastropods. Furthermore, several species of apple snails, especially *P. canaliculata* and *P. maculata*, are notorious invasive species in Asia and Hawaii, where they cause dramatic agricultural losses [10, 13], and other conservation concerns such as reductions in aquatic plant diversity and shift in wetland ecosystem functions [14, 15]. Therefore, there is substantial interest in the mechanisms of adaptation that have enabled these species to become invasive pests [16, 17], and in their biological control [18, 19].

Ampullariids are well-known for their diverse reproductive behaviours. While they are all dioecious and most genera of apple snails deposit their eggs in a jelly mass underwater, two genera (i.e., *Pomacea* and *Pila*) produce calcareous egg clutches that are deposited above the waterline. The shift from aquatic to aerial oviposition thus has occurred at least twice in the evolution of ampullariids, indicating parallel evolution in the genera *Pomacea* and *Pila* with respect to the changes in egg deposition behaviour and morphology (e.g., larger lung size and longer siphons [10]). Such behavioural and morphological adaptations in *Pomacea* are known to be accompanied by biochemical adaptations to predation [20]. In this regard, studies of several *Pomacea* species have shown that the major proteins of the egg perivitelline fluid (PVF), the fluid that surrounds and nourishes the embryo, possess multiple protective functions against predators including several anti-predation proteins (perivitellins) displaying anti-digestive, anti-nutritive, neurotoxic and aposematic properties [20–23]. Comparison between the protein-coding genes of *P. canaliculata* and *P. maculata* has revealed the involvement of gene duplication and positive selection in the formation and evolution of some PVF proteins [24, 25]. Further comparison with more distantly related genera/species would yield novel insights into the origin and evolution of PVF proteins that may underlie the diversity of reproductive behaviour and morphology in apple snails.

Apart from their use in ecological and evolutionary studies, some ampullariids, including *P. canaliculata* and *M. cornuarietis*, have been used in toxicological studies due to their high fecundity and the high sensitivity of their juveniles to pollutants such as heavy metals [26], organic pesticides [27] and organotins [28]. Mortality and deficiencies of growth or development have typically been considered to be informative toxicity end-points. Nevertheless, the lack of extensive genomic resources hinders the documentation of molecular pathways in toxicological studies of apple snails.

**Fig. 1:**
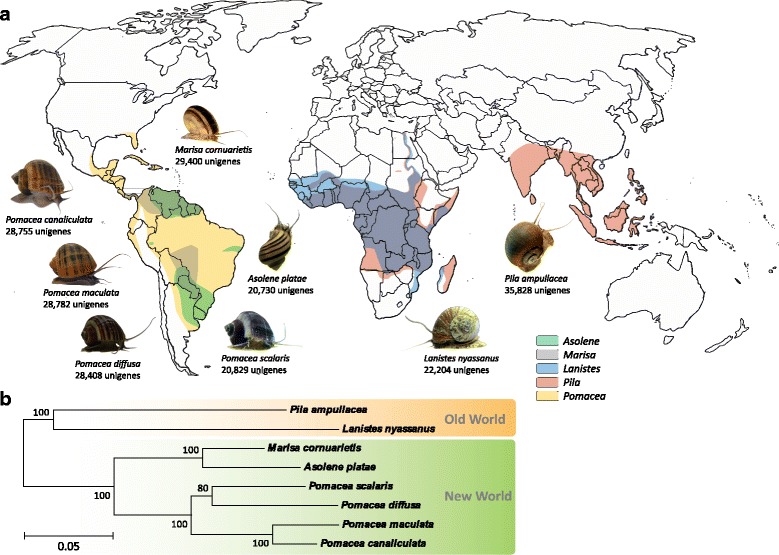
Geographical distribution and phylogeny of apple snails used in the present study. **a** Rough native distribution ranges of the Old World *(Lanistes* and *Pila*) and New World (*Asolene*, *Marisa* and *Pomacea*) genera/species [7, 56]. **b** A maximum likelihood tree showing the phylogenetic relationship among the eight species of ampullariids based on sequences of three genes used in previous phylogenetic studies of ampullariids [6, 52]. Methodological details for the phylogenetic analysis can be found in Additional file 1. Bootstrap support values are shown, as is a scale bar of 0.05 substitution per site. Photo credit: *L. nyassanus*, *Pila ampullacea* and *M. cornuarietis* (JCHI); *A. platae* and *P. scalaris* (SI); *P. canaliculata*, *P. maculata* and *P. diffusa* (HM)

To facilitate molecular-oriented studies on apple snails, we sequenced the transcriptomes of seven species of apple snails: *Lanistes nyassanus*; *Pila ampullacea*; *Asolene platae*; *Marisa cornuarietis*; *Pomacea diffusa*; *Pomacea scalaris* and *Pomacea canaliculata*. Together with our previously generated RNA-Seq data for *P. canaliculata* [29] and *P. maculata* [25], we cover eight species which represent five genera (Fig. 1a) and both the New World and Old World clades. Among the Old World species are *L. nyassanus*, a species endemic to Lake Malawi in the East African Rift [9, 30]; and *Pila ampullacea*, a common species in the paddy fields and irrigation channels of northern Thailand [31]. Among the New World species, *A. platae* is restricted to the La Plata River basin and has a distribution range from Bolivia to the northern Buenos Aires province of Argentina [32]; this species has a slower growth rate and smaller reproductive output than other ampullariids and probably less invasive [33]. The other five species have been introduced from South America to various freshwater ecosystems in North America, Asia and Pacific islands including Hawaii [10, 13, 34, 35]. Following their introduction, two species of *Pomacea* (i.e., *P. canaliculata* and *P. maculata*) have become widely distributed and they are regarded as some of the most notorious invasive species in freshwater habitats [7, 34, 36, 37]. Our species selection thus covers the various phylogenetic lineages, the diversity of reproductive strategies, the most important invaders, and members that are commonly adopted in ecotoxicology. Fig. 1b shows the phylogenetic relationships among the species used in this study, whereas a phylogeny featuring more extensive taxon sampling is presented in Additional files 1 and 2.

## Construction and content

### Sample collection and preparation

Adult snails were collected from the field in various regions of South America, Africa and Asia, or purchased from an aquarium shop in Hong Kong (Table 1). All snails were reared in aquaria filled with tap water and acclimated for at least one month at 26 ± 1 °C and a photoperiod of 14 h light/ 10 h dark. Snails were fed with a mixed diet of lettuce, carrot and fish meal once a day and the water was renewed twice a week. For most of the species, four to five female and male snails were chosen for dissection to obtain various tissues. For *L. nyassanus*, however, due to limited individuals available, only a female was used for dissection. Dissected tissues were immediately fixed in RNA*later*™ (Invitrogen, USA) and then stored at − 20 °C until they were subjected to RNA extraction.

**Table 1:** A summary of transcriptome data from eight apple snails used for database construction. Tissues: albumen gland (AG), digestive gland (DG), foot (F), gill (G), lung (L), mantle (M), kidney (K), stomach (S), testis (T) and other tissues (OT; including DG, F, M and T)

Species (SRA accession No.)	Sampling location	Tissue	Platform	Length (bp)	Clean read (bp)	Q20 (%)	GC (%)
Old World							
*Lanistes nyassanus Dohrn, 1865* (SRP127201)	F4 or F5 offspring from a lab inbred population; originally collected from Lake Malawi, Africa	AG	Hiseq2000	100	36,892,514	97.89	47.34
		OT without T	Hiseq2000	100	39,555,832	98.04	45.12
*Pila ampullacea* (Linnaeus, 1758) (SRP127221)	Wild-caught from Nong Phok District, Roi Et Province, Thailand	AG	Hiseq4000	100	78,216,048	98.66	46.44
		OT	Hiseq4000	100	82,268,586	98.76	44.34
New World							
*Asolene platae* (Maton, 1809) (SRP127224)	Wild-caught from Lago de Regatas, Buenos Aires, Argentina	AG	Hiseq2000	90	47,404,352	96.8	46.08
		AG	Hiseq4000	100	69,830,648	98.89	45.95
		OT without T	Hiseq4000	100	97,420,524	99.18	45.42
*Marisa cornuarietis* (Linnaeus, 1758) (SRP127203)	Aquarium shop, Mong Kok, Hong Kong	AG	Hiseq2000	90	51,889,926	97.55	46.11
		OT	Hiseq2000	90	53,590,040	96.62	45.24
*Pomacea diffusa* Blume, 1957 (SRP127204)	Aquarium shop, Mong Kok, Hong Kong	AG	Hiseq2000	90	54,266,010	97.71	44.11
		OT	Hiseq2000	90	54,579,594	96.91	44.91
*Pomacea scalaris* (d’Orbigny, 1835) (SRP127220)	Wild-caught from Lago de Regatas, Buenos Aires, Argentina	AG	Hiseq2000	90	72,341,892	98.43	43.05
*Pomacea canaliculata* (Lamarck, 1819) (SRP127216)	Wild-caught from Sheung Shui, Hong Kong	AG	Hiseq2500	125	50,399,554	97.90	45.04
		DG	Hiseq2500	125	45,063,414	97.78	49.34
		F	Hiseq2500	125	54,307,040	98.17	43.78
		G	Hiseq2500	125	49,217,508	98.01	45.20
		K	Hiseq2500	125	50,518,406	98.04	45.33
		L	Hiseq2500	125	40,886,322	97.97	45.30
		M	Hiseq2500	125	48,951,426	98.09	46.47
		S	Hiseq2500	125	44,860,264	97.65	45.28
		T	Hiseq2500	125	52,304,178	97.70	45.71
(SRA030614.2)	Wild-caught from Yuen Long, Hong Kong	OT without T	Hiseq2000	90	25,723,522	95.65	46.83
*Pomacea maculata* Perry, 1810 (SRP127219)	Wild-caught from Paraná River, Argentina	AG	Hiseq2000	100	52,732,156	98.20	44.94
		OT	Hiseq2000	100	54,961,478	98.26	45.05

### RNA isolation and sequencing

Total RNA was extracted separately from each tissue sample using TRIzol® reagent (Invitrogen, MA, USA) following the manufacturer’s protocol. In general, two RNA samples, including one of the albumen gland (AG), and one of other tissues (OT), which contained equal amounts of RNA extracted from three to four tissue types, were prepared for sequencing (Table 1). AG was always processed separately, because this organ, which secrets the perivitelline fluid that protects and nourishes the embryo, is expected to play a crucial role in the reproduction and evolution of ampullariids [24, 25, 38]. More tissue types of *P. canaliculata* were sequenced due to the need for producing tissue-specific gene expression data in another project for this species. To enhance the comprehensiveness of the transcripts for *P. canaliculata*, we combined our new data with the transcriptome data we generated from a previous study [29] for assembly. The transcriptome data of *P. canaliculata* from another study [39] were not included here because of uncertainty of sample preparation, and because more data would not likely improve the assembly metrics [40]. Raw reads of *P. maculata* were obtained from a recent publication [25], and re-assembled as described below. In *P. scalaris*, only AG was sequenced due to the lack of high quality RNA in OT preserved in RNA*later*. For all samples, the quality of extracted RNA was determined using an Agilent 2100 Bioanalyzer (Agilent Technologies, Germany). Samples with an RNA Integrity Number ≥ 8 were used for cDNA library construction using a TruSeq RNA Sample Prep Kit v2 (Illumina, California, USA), and sequenced in paired-end mode on an Illumina HiSeq sequencer (Illumina, California, USA). Library construction and sequencing were conducted by BGI Hong Kong as a commercial service.

### Transcriptome assembly and annotation

Illumina raw reads were cleaned by removing adaptor sequences, reads with > 5% unknown “N” bases or > 20% bases with a quality score ≤ 10 (Table 1). Trimmomatic v0.33 was then used to further remove low quality reads with a quality score < 20 and a length < 40 base pairs (bp) [41]. For each species, clean reads from different tissue samples were pooled for de novo assembly using Trinity 2.2.0 under default settings [42]. The assembled transcripts (ranging from 126,582 to 388,329; Table 2) were clustered with CD-HIT-EST 4.6.6 to reduce redundancy using a threshold of 95% sequence similarity [43]. Open reading frames (ORFs) were predicted with TransDecoder 3.0.0 (https://transdecoder.github.io/) using a threshold of ≥100 amino acids. Only the single best ORF per transcript was retained. The longest ORF in each gene cluster was selected as the unigene. Expression levels were estimated as transcripts per kilobase million read (TPM) using Salmon 0.7.2 [44], and unigenes with TPM less than 0.5 were considered as non-expressed [25]. The level of completeness of our eight assembled transcriptomes was evaluated using BUSCO (benchmarking universal single-copy orthologs) v1.1b [45].

**Table 2:** Transcriptome assembly and annotation statistics. To avoid confusion between *Pomacea* and *Pila*, the latter taxon is not abbreviated as “*P.*”

Items	*L. nyassanus*	*Pila ampullacea*	*A. platae*	*M. cornuarietis*	*P. diffusa*	*P. scalaris*	*P. canaliculata*	*P. maculata*
De novo assembly								
Assembled bases	164,160,894	238,879,002	214,102,711	159,734,791	168,090,829	141,684,727	536,808,768	145,979,415
Assembled transcripts	152,931	277,864	203,935	187,959	204,576	126,582	499,932	200,397
Assembled unigenes	122,779	212,935	156,912	161,143	171,676	98,100	215,456	154,712
Clustered transcripts	129,455	221,653	165,023	161,069	173,606	105,046	355,408	154,700
Clustered unigenes	114,869	192,301	142,773	147,375	157,064	89,910	211,621	136,742
Unigenes (transcripts)	22,204 (29,317)	35,828 (46,232)	20,730 (28,927)	29,400 (35,994)	28,408 (36,112)	20,829 (28,847)	28,755 (57,048)	28,782 (35,063)
Unigene N50 (bp)	1740	1683	1803	1440	1485	1629	1509	1320
Unigene length (bp) - average (min - max)	1222 (300–31,476)	1182 (300–19,023)	1281 (300–15,984)	1054 (300–23,508)	1076 (300–25,756)	1163 (300–13,624)	1074 (300–40,192)	974 (300–17,707)
BUSCO								
Complete (%)	86.83	92.41	82.09	77.82	79.95	80.43	80.07	77.46
Fragmented (%)	4.15	3.68	4.74	13.52	11.63	8.78	7.47	12.81
Annotation (unigenes)								
NCBI nr	17,065 (76.86%)	27,254 (76.07%)	16,051 (77.43%)	22,579 (76.80%)	21,405 (75.35%)	16,705 (80.20%)	20,051 (69.73%)	21,625 (75.13%)
GO	10,697 (48.18%)	18,717 (52.24%)	9852 (47.53%)	14,274 (48.55%)	13,519 (47.59%)	10,394 (49.90%)	12,216 (42.48%)	13,671 (47.50%)
KEGG	3783 (17.04%)	5467 (15.26%)	3546 (17.11%)	4215 (14.34%)	4061 (14.30%)	3801 (18.25%)	3693 (12.84%)	4059 (14.10%)

Predicted protein sequences were annotated using BLASTp 2.4.0+ [46] against NCBI’s non-redundant (nr) database with an *E*-value of 1 × 10^− 5^. Gene Ontology (GO) function for each unigene was assigned using Blast2GO [47] with BLASTp nr input. Sequences were also submitted to the Kyoto Encyclopedia of Genes and Genomes (KEGG) Automatic Annotation Sever (http://www.genome.jp/kegg/kaas/) to determine their functional relationships using the bi-directional best-hit method. References for the KEGG annotation included the default representative eukaryotic genomes as well as the genomes of several invertebrates: *Helobdella robusta*, *Lottia gigantea*, *Crassostrea gigas*, *Octopus bimaculoides*, *Schistosoma mansoni*, *Nematostella vectensis* and *Hydra vulgaris*. The annotation results are summarized in Table 2.

### AmpuBase database construction

AmpuBase is a relational database that provides public access to these newly assembled ampullariid transcriptomes and annotations. The database structure and layout are similar to those of PcarnBase [48], except that data from several species can be searched at the same time and that the GO and KEGG search pages are integrated. In brief, for each species, a relational database was developed using MySQL v5.6.34 and hosted on an Apache HTTP server. The BLAST search function is powered by ViroBLAST [49] using the PHP programming language. The database consists of DNA and protein sequences of all unigenes that are linked with associated NCBI nr, GO and KEGG annotations through unigene ID. The database consists of five entity tables (“NCBI annotation”, “Proteins”, “DNAs”, “Gene Ontology” and “KEGG”) and two relation tables (“NCBI_GO_relation” and “NCBI_KEGG_relation”).

## Utility and discussion

### Transcriptome assembly metrics

There were between 72,341,892 to 462,231,634 bp of clean data, corresponding to between 20,730 and 35,828 unigenes with ORFs in each of the eight species (Table 2; Fig. 1a). The mean N50 value (shortest sequence length at 50% of the unigenes; 1576 bp) and the percentage of annotated unigenes (average 75.9%) in our study are higher than the corresponding values from previously published ampullariid transcriptomes (*P. canaliculata,* N50: 283 bp, 29.2% unigenes annotated [29]; *P. maculata,* N50: 1332 bp, 36.6% unigenes annotated [25]). Our transcriptome assembly metrics are comparable to those of recently published transcriptomes from other families of mollusks (Table 3), indicating the overall robustness of our transcriptome sequencing, assembly and annotation pipeline.

**Table 3:** Comparison of transcriptome assembly metrics between this study and some other studies of mollusks

Items	This study (mean)	*P. canaliculata* [our previous study; [29]	*P. maculata* [our previous study; [25]	*Reishia clavigera* [57]	*Potamopyrgus antipodarum* [58]	*Lottia* cf. *kogamogai* [59]	*Nucula tumidula* [59]	*Mytilisepta virgata* [60]
De novo assembly								
transcripts	37,193	128,436	105,349	38,466	62,862	34,794	273,272	49,501
Unigenes	26,867	–	–	32,798	–	–	–	–
N50 (bp)	1576	283	1332	2236	690	817	2100	1046
Mean length (bp)	1128	420	878	1709	999	–	–	679
BUSCO								
Complete genes	82.13%	40.21%	71.89%	93.00%	89.09%	33.93%	83.63%	66%
Fragmented	8.35%	39.38%	18.86%	3.56%	6.80%	34.48%	11.39%	10%
Annotation								
Protein database	75.95%	24.04%	33.79%	74.40%	25.13%	48.23%	14.11%	25%
GO	48.00%	6.83%	15.30%	45.42%	(overall)	25.22%	8.75%	(overall)
KEGG	15.41%	10.07%	23.61%	15.66%		27.04%	6.78%	

To further evaluate the completeness of transcriptomes, we examined the proportions of complete as well as partial homologs of 843 conserved metazoan genes within the eight coding unigene sets. The transcriptomes contain 77.46 to 92.41% of the complete conserved metazoan genes, and 87.54 to 96.09% of the genes if fragmented BUSCO hits are also included (Table 2). These BUSCO metrics are comparable with those of other mollusc transcriptomes published in recent years (Table 3).

### AmpuBase: Functions and applications

**Fig. 2:**
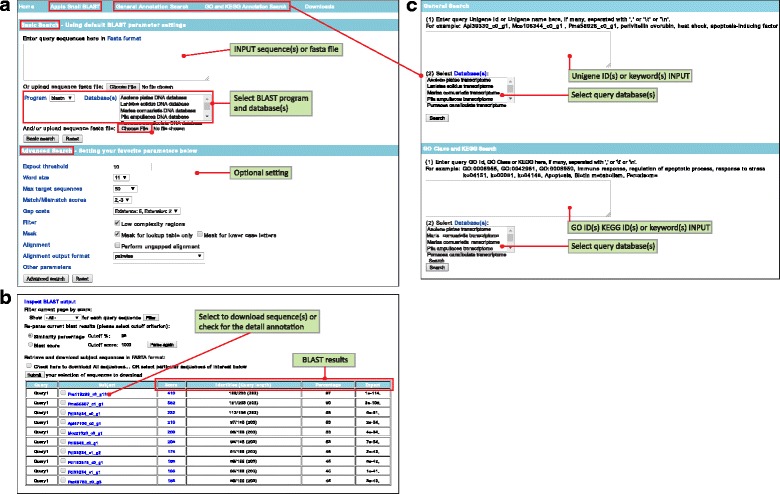
The web interface of AmpuBase. **a** Illustration of the Basic and Advanced BLAST search options. **b** An example of the search result of a BLAST search, showing matched sequences, each with their BLAST statistics. **c** Illustration of the search functions in AmpuBase based on annotation

AmpuBase is available online via web interface at http://www.comp.hkbu.edu.hk/~db/AmpuBase/. The database can be searched with BLAST or other query terms. The BLAST search function allows users to blast query sequence(s) or fasta files against single or multiple DNA/protein datasets with default settings (under Basic Search option) or customizable settings (under Advance Search option) (Fig. 2a). Upon submitting a BLAST search, matched sequences are returned with their *E*-value and similarity score, and information on the corresponding annotation can be obtained by clicking “Unigene ID” (Fig. 2b).

Apart from BLAST search, the transcriptome data can be searched in two other ways (Fig. 2c). General Annotation Search allows one to query the relevant annotations (i.e., NCBI annotation, GO and KEGG) either by using the unigene ID or unigene name (e.g., perivitellin ovorubin). Each successful query returns a table that contains Unigene ID, NCBI’s nr, GO and KEGG description (if available). The resultant sequences can be downloaded by selecting the Unigene ID and clicking “Submit” for further analysis, for example, phylogenetic analysis of perivitellin ovorubins, major and multiple functional proteins in PVF [20, 24, 25]. In addition, GO and KEGG Annotation Search is also provided for searching GO and KEGG information using GO ID, KEGG ID or a keyword. All sequence data for these ampullariid transcriptomes are available for download under the “Downloads” menu, for transcriptome wide data mining and analysis of a specific gene.

## Conclusions

In this study, we have generated a large set of transcriptome data for eight species that represent five genera of Ampullariidae. These data are compiled in a relational database, AmpuBase, which greatly enhances the publicly available genomic resources for ampullariids. The database provides tools for sequence- or keyword-based query functions, which will facilitate in-depth ecological and evolutionary studies on ampullariids, and comparative studies with other invertebrates. AmpuBase will be updated when more genomic data become available in the future.

## Additional file

Additional file 1: Phylogenetic tree of ampullariids based on DNA sequences of cytochrome *c* oxidase I (COI), 16S rRNA (16S) and 18S rRNA (18S) as listed in Additional file 2. Sequences were aligned and gaps were trimmed with MUSCLE. Phylogenetic analysis was conducted using the concatenated sequences (COI: 502 bp; 16S: 362 bp; 18S: 269 bp). The maximum-likelihood method implemented in MEGA5 [50] was used and the GTR + Γ + I evolutionary model was selected. Members of Viviparidae and Campanilidae served as outgroups. Values at nodes are percentages of 100 bootstrap replicates. Scale bar represents 0.1 substitution per site. Species with transcriptomes assembled in the present study are highlighted in blue. List of taxa and GenBank accession numbers for sequences of COI, 16S and 18S used in phylogenetic analysis. (DOCX 364 kb)

Additional file 2: List of taxa and GenBank [51] accession numbers for sequences of COI, 16S and 18S used in phylogenetic analysis [6, 52–55]. (DOCX 20 kb)

## Abbreviations

16S: 16S rRNA
18S: 18S rRNA
AG: Albumen gland
bp: Base pair
BUSCO: Benchmarking universal single-copy orthologs
COI: Cytochrome *c* oxidase I
DG: Digestive gland
F: Foot
G: Gill
GO: Gene Ontology
K: Kidney
KEGG: Kyoto Encyclopedia of Genes and Genomes
L: Lung
M: Mantle
N50: Shortest sequence length at 50% of the unigenes
nr: Non-redundant
ORFs: Open reading frames
OT: Other tissues
PVF: Perivitelline fluid
S: Stomach
T: Testis
TPM: Transcripts per kilobase million read
