# Supplementary material for: AmpuBase: a transcriptome database for eight species of apple snails (Gastropoda: Ampullariidae)
Source: BMC Genomics. 2018 Mar 5;19:179. doi: 10.1186/s12864-018-4553-9 (PMC5839033; doi:10.1186/s12864-018-4553-9)
Supplement: Supplementary file 1 — Phylogenetic tree of ampullariids based on DNA sequences of cytochrome c oxidase I (COI), 16S rRNA (16S) and 18S rRNA (18S) as listed in Additional file 2. Sequences were aligned and gaps were trimmed with MUSCLE. Phylogenetic analysis was conducted using the concatenated sequences (COI: 502 bp; 16S: 362 bp; 18S: 269 bp). The maximum-likelihood method implemented in MEGA5 [50] was used and the GTR + Γ + I evolutionary model was selected. Members of Viviparidae and Campanilidae served as outgroups. Values at nodes are percentages of 100 bootstrap replicates. Scale bar represents 0.1 substitution per site. Species with transcriptomes assembled in the present study are highlighted in blue. List of taxa and GenBank accession numbers for sequences of COI, 16S and 18S used in phylogenetic analysis. (DOCX 364 kb) [file 12864_2018_4553_MOESM1_ESM.docx]

**Additional file 1**
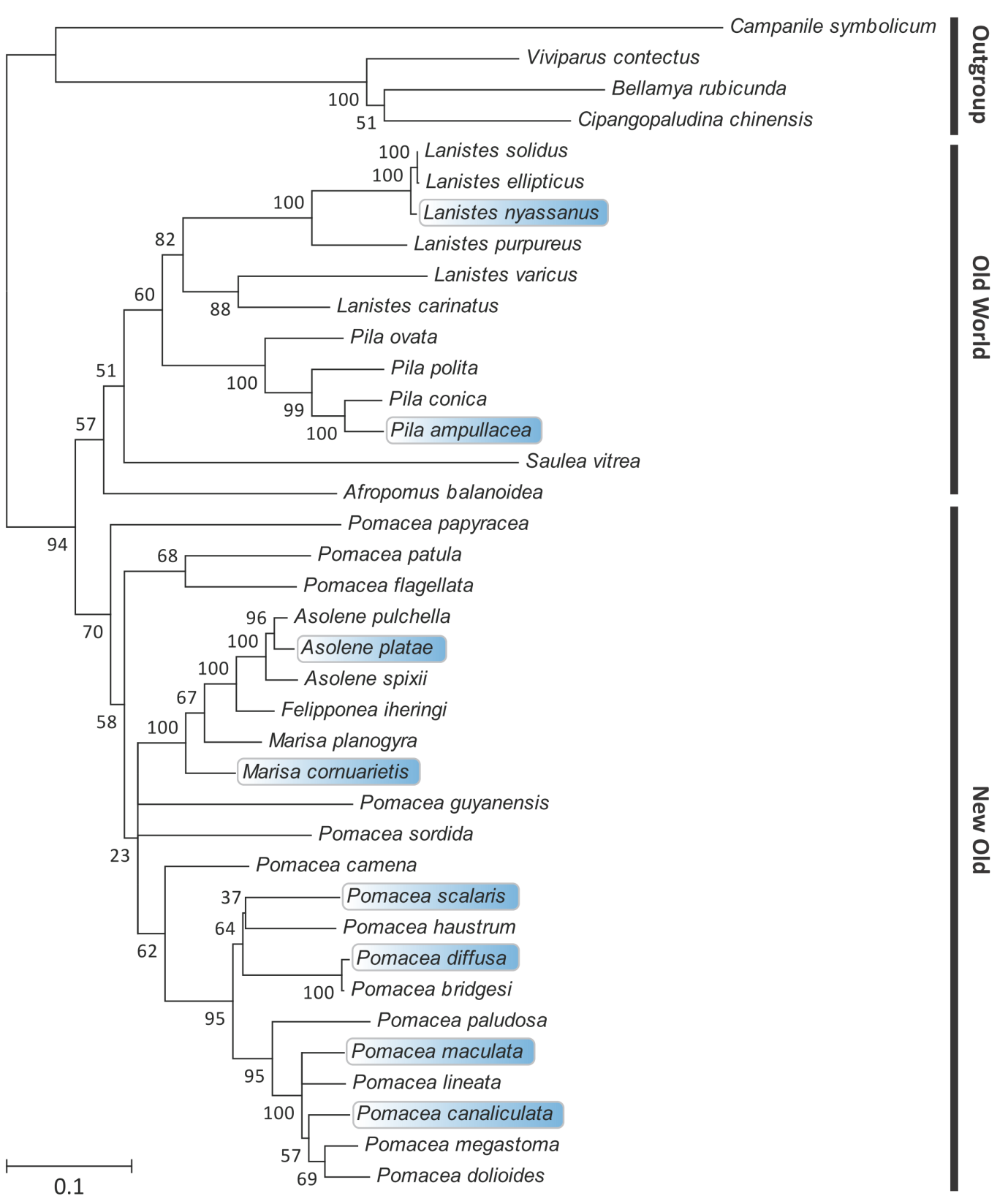


Phylogenetic tree of ampullariids based on DNA sequences of cytochrome *c* oxidase I (COI), 16S rRNA (16S) and 18S rRNA (18S) as listed in Additional file 2. Sequences were aligned and gaps were trimmed with MUSCLE. Phylogenetic analysis was conducted using the concatenated sequences (COI: 502 bp; 16S: 362 bp; 18S: 269 bp). The maximum-likelihood method implemented in MEGA5 [56] was used and the GTR + Γ + I evolutionary model was selected. Members of Viviparidae and Campanilidae served as outgroups. Values at nodes are percentages of 100 bootstrap replicates. Scale bar represents 0.1 substitution per site. Species with transcriptomes assembled in the present study are highlighted in blue.

**Reference**

1. Tamura K, Peterson D, Peterson N, Stecher G, Nei M, Kumar S. MEGA5: molecular evolutionary genetics analysis using maximum likelihood, evolutionary distance, and maximum parsimony methods. Mol Biol Evol. 2011;28:2731-2739.
