# Supplementary material for: AmpuBase: a transcriptome database for eight species of apple snails (Gastropoda: Ampullariidae)
Source: BMC Genomics. 2018 Mar 5;19:179. doi: 10.1186/s12864-018-4553-9 (PMC5839033; doi:10.1186/s12864-018-4553-9)
Supplement: Supplementary file 2 — List of taxa and GenBank [51] accession numbers for sequences of COI, 16S and 18S used in phylogenetic analysis [6, 52–55]. (DOCX 20 kb) [file 12864_2018_4553_MOESM2_ESM.docx]

List of taxa and GenBank [57] accession numbers for sequences of COI, 16S and 18S used in phylogenetic analysis [6, 39, 58-60].

| **Species** | **16S** | **18S** | **COI** |
| --- | --- | --- | --- |
| **Outgroup** |  |  |  |
| *Bellamya rubicunda* | EU274483.1 | EU274535.1 | EU274556.1 |
| *Campanile symbolicum* | AY010507.1 | DQ916524.1 | AY296828.1 |
| *Cipangopaludina chinensis* | FJ710213.1 | FJ710250.1 | EU528474.1 |
| *Viviparus contectus* | EU274484.1 | EU274536.1 | EU274557.1 |
| **Old World** |  |  |  |
| *Afropomus balanoidea* | FJ710215.1 | FJ710252.1 | FJ710299.1 |
| *Lanistes carinatus* | EU274486.1 | EU274537.1 | EU274560.1 |
| *Lanistes ellipticus* | EU274487.1 | EU274538.1 | EU274561.1 |
| *Lanistes nyassanus* | EU274488.1 | EU274539.1 | EU274562.1 |
| *Lanistes purpureus* | EU274490.1 | EU274542.1 | EU274565.1 |
| *Lanistes solidus* | EU274491.1 | EU274543.1 | EU274566.1 |
| *Lanistes varicus* | EU274492.1 | EU274544.1 | EU274567.1 |
| *Pila ampullacea* | EU274494.1 | EU274546.1 | EU274569.1 |
| *Pila conica* | EU274495.1 | EU274547.1 | EU274570.1 |
| *Pila ovata* | EU274496.1 | EU274548.1 | EU274571.1 |
| *Pila polita* | EU274499.1 | EU274551.1 | EU274572.1 |
| *Saulea vitrea* | FJ710217.1 | EU274555.1 | EU274575.1 |
| **New World** |  |  |  |
| *Asolene platae* | FJ710224.1 | FJ710262.1 | EU528517.1 |
| *Asolene pulchella* | FJ710225.1 | FJ710263.1 | FJ710306.1 |
| *Asolene spixii* | FJ710226.1 | FJ710264.1 | FJ710307.1 |
| *Felipponea iheringi* | FJ710227.1 | FJ710265.1 | FJ710308.1 |
| *Marisa cornuarietis* | EU274493.1 | EU274545.1 | EF514953.1 |
| *Marisa planogyra* | FJ710223.1 | FJ710260.1 | EU528541.1 |
| *Pomacea bridgesi* | EU274500.1 | AF046057.1 | DQ093524.1 |
| *Pomacea camena* | FJ710245.1 | FJ710293.1 | EF515059.1 |
| *Pomacea canaliculata* | FJ710234.1 | EU274554.1 | EU528529.1 |
| *Pomacea diffusa* | FJ710242.1 | FJ710283.1 | FJ710317.1 |
| *Pomacea dolioides* | FJ710232.1 | FJ710271.1 | EU528500.1 |
| *Pomacea flagellata* | FJ710247.1 | FJ710295.1 | FJ710327.1 |
| *Pomacea guyanensis* | FJ710243.1 | FJ710287.1 | FJ710319.1 |
| *Pomacea haustrum* | FJ710239.1 | FJ710280.1 | EU528592.1 |
| *Pomacea lineata* | FJ710230.1 | FJ710268.1 | FJ710309.1 |
| *Pomacea maculata* | FJ710228.1 | FJ710266.1 | EU528559.1 |
| *Pomacea megastoma* | FJ710249.1 | FJ710297.1 | FJ710324.1 |
| *Pomacea paludosa* | FJ710237.1 | FJ710278.1 | FJ710329.1 |
| *Pomacea papyracea* | FJ710248.1 | FJ710296.1 | EU528590.1 |
| *Pomacea patula* | FJ710246.1 | FJ710294.1 | FJ710328.1 |
| *Pomacea scalaris* | FJ710240.1 | FJ710281.1 | FJ710326.1 |
| *Pomacea sordida* | FJ710244.1 | FJ710291.1 | FJ710316.1 |

**References**

1. Tamura K, Peterson D, Peterson N, Stecher G, Nei M, Kumar S. MEGA5: molecular evolutionary genetics analysis using maximum likelihood, evolutionary distance, and maximum parsimony methods. Mol Biol Evol. 2011;28:2731-2739.
2. Benson DA, Cavanaugh M, Clark K, Karsch-Mizrachi I, Ostell J, Pruitt KD, Sayers EW. GenBank. Nucleic Acids Res. 2017.
3. Colgan D, Ponder W, Beacham E, Macaranas J. Molecular phylogenetics of Caenogastropoda (Gastropoda: Mollusca). Mol Phylogenet Evol. 2007;42:717-737.
4. Lydeard C, Holznagel WE, Glaubrecht M, Ponder W. F. Molecular phylogeny of a circum-global, diverse gastropod superfamily (Cerithioidea: Mollusca: Caenogastropoda): pushing the deepest phylogenetic limits of mitochondrial LSU rDNA sequences. Mol Phylogenet Evol. 2002;22:399-406.
5. Giribet, G. et al. Evidence for a clade composed of molluscs with serially repeated structures: monoplacophorans are related to chitons. Proc Natl Acad Sci USA. 2006;103:7723-7728.
